# Maternity care clinician inclusion in Medicaid Accountable Care Organizations

**DOI:** 10.1371/journal.pone.0282679

**Published:** 2023-03-08

**Authors:** Michael I. Cooper, Laura B. Attanasio, Kimberley H. Geissler

**Affiliations:** 1 Department of Health Promotion and Policy, School of Public Health and Health Sciences, University of Massachusetts Amherst, Amherst, Massachusetts, United States of America; 2 Tufts University School of Medicine, Boston, Massachusetts, United States of America; University of Palermo: Universita degli Studi di Palermo, ITALY

## Abstract

**Background:**

Medicaid Accountable Care Organizations (ACO) are increasingly common, but the network breadth for maternity care is not well described. The inclusion of maternity care clinicians in Medicaid ACOs has significant implications for access to care for pregnant people, who are disproportionately insured by Medicaid.

**Purpose:**

To address this, we evaluate obstetrician-gynecologists (OB/GYN), maternal-fetal medicine specialists (MFM), certified nurse midwives (CNM), and acute care hospital inclusion in Massachusetts Medicaid ACOs.

**Methodology/Approach:**

Using publicly available provider directories for Massachusetts Medicaid ACOs (n = 16) from December 2020 –January 2021, we quantify obstetrician-gynecologists, maternal-fetal medicine specialists, CNMs, and acute care hospital with obstetric department inclusion in each Medicaid ACO. We compare maternity care provider and acute care hospital inclusion across and within ACO type. For Accountable Care Partnership Plans, we compare maternity care clinician and acute care hospital inclusion to ACO enrollment.

**Results:**

Primary Care ACO plans include 1185 OB/GYNs, 51 MFMs, and 100% of Massachusetts acute care hospitals, but CNMs were not easily identifiable in the directories. Across Accountable Care Partnership Plans, a mean of 305 OB/GYNs (median: 97; range: 15–812), 15 MFMs (Median: 8; range: 0–50), 85 CNMs (median: 29; range: 0–197), and half of Massachusetts acute care hospitals (median: 23.81%; range: 10%-100%) were included.

**Conclusion and practice implications:**

Substantial differences exist in maternity care clinician inclusion across and within ACO types. Characterizing the quality of included maternity care clinicians and hospitals across ACOs is an important target of future research. Highlighting maternal healthcare as a key area of focus for Medicaid ACOs–including equitable access to high-quality obstetric providers–will be important to improving maternal health outcomes.

## Introduction

Accountable Care Organizations (ACOs) are a payment and delivery model designed to incentivize the provision of high-quality care at lower cost. Evidence of reduced spending in Medicare ACOs compared to traditional payment models has prompted states to experiment with the use of ACOs in their Medicaid programs [1–4]. Preliminary reports from Medicaid ACOs have shown a reduction in emergency department use, rehospitalizations, and readmissions [5]. Medicaid ACOs are increasingly common, with active programs in 12 states [6].

Distinct in structure from Medicare ACOs where Medicare enrollees with traditional Medicare assigned to ACOs can visit any primary care or specialist clinician accepting Medicare [7], Medicaid ACO enrollees are generally restricted to primary care and specialist clinicians within the ACO and/or associated managed care plan [8]. Given that Medicaid enrollees can face access issues related to a lack of participating clinicians [9], understanding the clinicians included for members of Medicaid ACOs is an important policy question. Federal government standards require states to have quantitative network adequacy standards (e.g., time and distance standards) [10], but these differ substantially in measurement and enforcement by state [11]. Thus, even provider networks for Medicaid managed care plans and Medicaid ACOs that meet state government regulations may vary substantially and impact patient access [10].

As of 2020, over half of physicians nationally are participating in at least one ACO of any type with just over a quarter of physicians participating in a Medicaid ACO [12]. Physicians in hospital-owned practices, multispecialty groups, and in practices with at least some primary care physicians are more likely to participate in Medicaid ACOs [13]. Previous research on novel Medicaid ACO models has identified barriers to provider participation, including provider perception of potential penalization for their performance as well as lack of incentives for individual clinicians to change their delivery model [14].

Most research on clinician participation in Medicaid ACOs has focused on primary care clinicians [2], but the inclusion of maternity care providers–both clinicians and hospitals with obstetric departments–is also critical for a Medicaid population that is disproportionately likely to use these services. Due to higher income eligibility thresholds during pregnancy, over 40% of births nationally are covered by Medicaid [15, 16]. Recent work identifying influential characteristics in women’s choice of where to receive obstetric/gynecologic or reproductive care ranked insurance network as the second most important factor, behind quality [17]. Another study found 96% of mothers cited accepting their health insurance plan as a major factor when choosing a prenatal care provider and birth hospital [18, 19]. Understanding inclusion of providers and overlap among Medicaid ACOs is particularly important given ongoing transitions in insurance plans, including among those insured by Medicaid, during the perinatal period [20]. Obstetrician-gynecologists (OB/GYNs) are uniquely placed as physicians because they provide preventive services and act as primary care providers for some patients and 25% of women consider their OB/GYN to be their primary care provider; however, they do not always provide the full range of preventive services offered by family physicians and internists, and have not been used to build primary care-focused ACOs [21–26]. However, OB/GYNs differ from other specialists in that some states and state Medicaid programs require health plans to allow patients to see OB/GYNs without a referral, even if they are not the patient’s designated PCP [19].

As alternative payment models, including ACOs, become more common in all insurance types, understanding the differences in provider network breadth in Medicaid ACOs is increasingly important to ensure patient access to care. In Medicaid ACOs, adequate networks of OB/GYN providers are essential to ensuring high quality care for women. In this study, we quantify maternity care provider inclusion in Massachusetts Medicaid ACOs, implemented starting in March 2018, and analyze differences in provider inclusion by specific Medicaid ACO type.

## Methods

### Overview

Using publicly available provider directories for Massachusetts Medicaid ACOs and data on provider supply, we quantified inclusion of obstetrician-gynecologists (OB/GYN), maternal-fetal medicine (MFM) specialists, certified nurse-midwives (CNM), and acute care hospitals with obstetrics departments in Medicaid ACOs. We then compared the number and type of providers across ACO types, across ACOs within ACO type, and to the total number of providers within Massachusetts.

In Massachusetts, Medicaid ACO models were implemented starting in March 2018 with two ACO distinct models available as of 2021 [27, 28]. The first model, Accountable Care Partnership Plans (ACPP), operates within a specific service area and restricts the provider network to those within the contracted Managed Care Organization. The second model, Primary Care ACOs (PCACO), rely on specific in-network primary care providers, but provide access to the entire Medicaid specialist and hospital network [29]. Both models consist of a two-sided risk payment structure in which the ACO can either receive a greater portion of savings for high value care, or pay a penalty to Medicaid if the cost of care exceeds predefined targets [28]. PCACOs are only eligible to receive shared savings if they reach a minimum benchmark on quality metrics, including prenatal care metrics [30]. ACPP ACOs have similar quality metrics factored into the shared risk payment arrangements to provide accountability [31]. As of January 2021, over three quarters of Medicaid enrollees’ primary coverage is through an ACO [32]. Among ACO enrollees, 60% are enrolled in an Accountable Care Partnership Plan and 40% are enrolled in a Primary Care ACO [32]. This study used publicly available information and did not include data on human subjects. Therefore, Institutional Review Board approval was not necessary.

### Data

We analyzed obstetric provider inclusion in the 13 ACPP ACOs and three PCACOs operating in Massachusetts Medicaid in January 2021. We analyzed the inclusion of OB/GYNs, MFMs, and CNMs using ACO provider directories available online between December 2020 and January 2021. Five ACPP ACOs provided print directories generated directly from online directories located on the ACO websites. Eight ACPP ACOs provided separate, distinct printed directories available on the ACO websites. The three PCACOs utilized the MassHealth provider network for specialists, and we used the MassHealth online directory for these ACOs. Enrollment data for each ACO is determined as of July 2018 [28].

For ACPP plans, clinicians included in the analysis were those listed in the categories of obstetrician, obstetrician/gynecology, gynecology, or maternal-fetal medicine within the provider directories. For certified nurse midwives (CNM), the directories which included CNMs had a separate section listing those who were included. For directories including a provider identification number (such as an NPI), the identifier was extracted, and duplicates were removed to determine the total number of providers included in that plan. For all others, duplicates were removed manually based on clinician name. If provider directories included practice names, we did not count these practices towards the number of included clinicians; we checked a number of these by hand in each directory to ensure that the majority of clinicians practicing in those organizations were included in the provider directory and discuss in the results where there were any deviations from this. We were not able to include family medicine physicians who provide maternity care due to the difficulty of systematically identifying these specific physicians.

For the PCACO plans, we searched the online provider directory by specialty for “Obstetrics (OB/GYN)” and included individual clinicians in the count. MFMs were not classified separately in the online provider directory but were included within OB/GYN provider listings. To determine MFM inclusion, MFM providers listed within the 2019 Massachusetts Registration of Provider Organizations Physician Roster (MA RPO) were manually identified within the covered Medicaid OB/GYN providers [33]. The MA RPO includes physicians practicing in organizations meeting certain patient and revenue thresholds, and may not include MFMs practicing independently or in small organizations. We then searched the directory using categorical drop down boxes of “nurse midwives” for CNMs. No duplicates by name appeared in these directories. We used the same procedure as above to exclude listed practices in this directory. The provider directory online notes, “Please Note: Some providers in this directory may no longer accept MassHealth. Before making an appointment, please contact the provider to confirm that they are accepting new MassHealth patients.”

The Medicaid waiver implementing the Medicaid ACOs includes a policy that PCACOs may designate a “referral circle” of ACO-preferred specialists that patients may visit without a required PCP referral [34]; however, enrollees still have access to the full MassHealth network. Publicly available information demonstrates only one of the three PCACOs states that they include a referral circle and none actually list providers included in the referral circle, so we do not analyze these further.

The total number of OB/GYNs and CNMs in Massachusetts for comparison to provider directories is based on estimates from the Area Health Resources File from the Health Resources and Services Administration [35]. We calculate the number of OB/GYNs as MDs and DOs within the Obstetrics and Gynecology health profession subcategory (2019–2020). The number of CNMs are from the nurse midwife category (2019–2020). The Area Health Resources File from the Health Resources and Services Administration does not offer an estimate of MFMs. Therefore, we utilize the 2019 Massachusetts Registration of Provider Organizations Physician Roster to estimate the total number of MFMs practicing in Massachusetts based on a primary or secondary specialty of Maternal-Fetal Medicine [33]. We compare the number of OB/GYNs and MFMs included in ACOs to the overall number of practicing OB/GYNs and MFMs in Massachusetts to contextualize the comprehensiveness of specialist networks.

We used the MassHealth Enrollment Guide (January 2019) to determine hospital inclusion for each ACO, limited to in-state acute care hospitals with an obstetrics department that conducts deliveries [29]. Acute care hospitals in Massachusetts were identified by those included in the Massachusetts’ Center for Health Information and Analysis (CHIA) Acute Hospitals Profiles [36]. Birth and obstetric department information was primarily determined by hospital profile data from the American Hospital Association (AHA) [37]. For any hospitals that did not have AHA data, data from the 2017 Massachusetts Birth Report [38] as well as individual hospital websites was used. The service areas for each ACO and hospital location was used to determine which hospital referral region(s) applied to specific ACOs and hospitals. MassHealth service areas and hospital locations were matched to hospital service areas and then subsequently to hospital referral regions. No service area has fewer than four ACOs to choose from, and some areas in Greater Boston and the South Shore have as many as 11 [39].

### Measures

The primary outcomes of this study are the number of 1) Maternity care clinicians including OB/GYN physicians, MFM physicians, and CNMs and 2) acute care hospitals with obstetrics departments included in each of the Massachusetts Medicaid ACO plan types.

### Analysis

Descriptive statistics for ACO provider inclusion were calculated; these statistics give equal weight to each ACO. Comparisons were made between ACO types and to the number of practicing physicians in MA. For ACPPs, which have different numbers of included physicians, we compare the number of included clinicians to the ACO enrollment. All analyses were conducted using Microsoft Excel.

## Results

Across Accountable Care Partnership Plan ACOs, there was substantial variation in number of providers included [Table 1]. The mean number of included OB/GYNs was 305, with a median of 97 and a range of 15 to 812 per ACO. ACPP ACOs included a mean of 85 CNMs, with a median of 29 CNMs and a range of zero to 197 per ACO. Although CNMs were well represented in most ACOs, two ACOs did not list any CNMs in their provider network.

**Table 1:** Descriptive statistics of maternity care clinician inclusion.

	ACO Type	Mean	Median	Minimum	Maximum
**OB/GYNs Statewide**					
	Accountable Care Partnership Plan	305	97	15	812
	Primary Care ACO	1,185	1,185	1,185	1,185
	Total Practicing OB/GYNs from AHRF**	1,046			
**MFMs Statewide**					
	Accountable Care Partnership Plan	15	8	0	50
	Primary Care ACO	51	51	51	51
	Total Practicing MFMs from MA RPO***	61			
**CNMs Statewide**					
	Accountable Care Partnership Plan	85	29	0	197
	Primary Care ACO	*	*	*	*
	Total Practicing Nurse Midwives from AHRF****	345			

**Notes:** * is used for Primary Care ACOs because no individual CNMs are listed in MassHealth directory. The directory listing for the category of nurse midwives includes practices, and physicians (not necessarily practicing in obstetrics or gynecology)

** Total Practicing OB/GYNs are MDs and DOs within the Obstetrics and Gynecology health profession subcategory (2019–2020)

***Total physicians identified with a primary or secondary specialty of a variant of Maternal-Fetal Medicine (2019)

****Total Practicing Nurse Midwives are nurse midwives within the Nurse Midwife with NPI profession subcategory (2019–2020).

Primary Care ACOs utilize the full Massachusetts Medicaid specialist network, and therefore all ACOs of this type have the same OB/GYN inclusion of 1185 OB/GYNs and MFM inclusion of 51 [Table 1]. In addition to these 1185 listed OB/GYNs, there are a large number of practices included in the OB/GYN category. However, when we investigated these practices more fully, many of the practices included do not have any OB/GYNs at the practice (e.g., an infectious disease practice), thus in line with the methodology did not include them towards the count of included clinicians. The online provider directory for the PCACOs did not list any individual CNMs when filtering for nurse midwives as described above. However, the provider directory nurse midwives category included practices (the majority were non obstetric/women’s health practices) and physicians (the majority of whom did not practice in obstetrics or gynecology).

We compared these included clinician numbers to the total practicing obstetric clinicians in Massachusetts according to the AHRF. The AHRF notes there are 1,046 OB/GYNs, which is higher than the number of OB/GYNs included in any ACPP provider networks; however, it is smaller than the number of OB/GYNs listed in the PCACO network. The number of practicing Maternal-Fetal Medicine from the MA RPO is 61. The number of practicing CNMs in Massachusetts from AHRF is 345.

**Fig 1:**
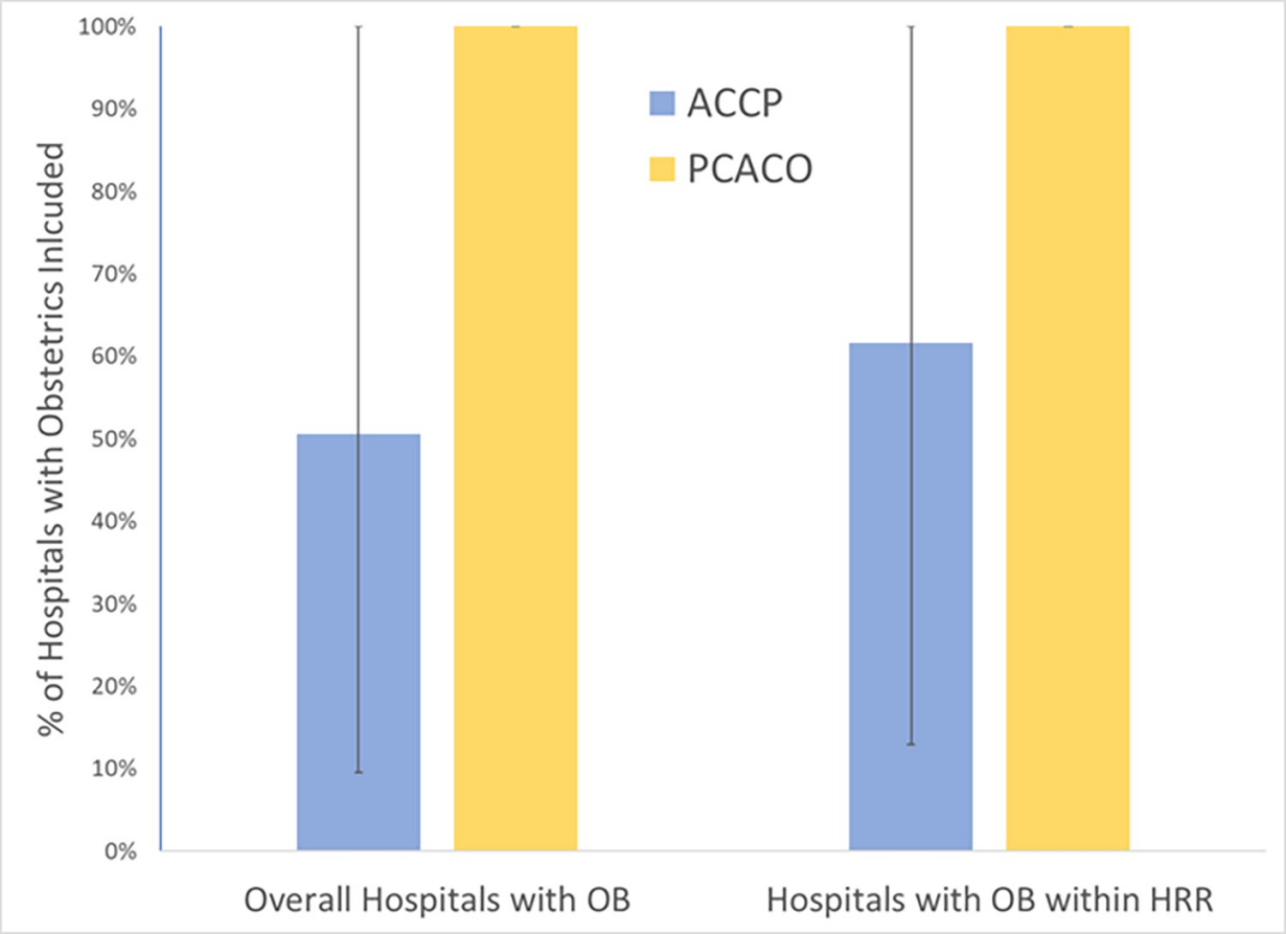
Percent of hospitals with obstetrics departments included within Medicaid ACOs, by ACO type. **Notes:** Vertical bars represent minimum and maximum values for ACOs within that category.

The percentage of hospitals with obstetrics departments included also varied substantially within and across ACO types [Fig 1]. Accountable Care Partnership plans had variation in number of included hospitals, with ACPP ACOs including a mean of 51% of acute care hospitals with an obstetric department in Massachusetts (range: 10%-100%). When limiting consideration to Massachusetts hospitals within the hospital referral region (HRR) of the service areas for the ACO, there was higher hospital inclusion (mean: 62%; range: 13%-100%). Five ACPP ACOs included all acute care hospitals in Massachusetts. However, three ACPP ACOs included fewer than 15% of Massachusetts hospitals within the HRR region(s) serviced. Primary Care ACO plans included 100% of acute care hospitals with an obstetric department in Massachusetts.

**Fig 2:**
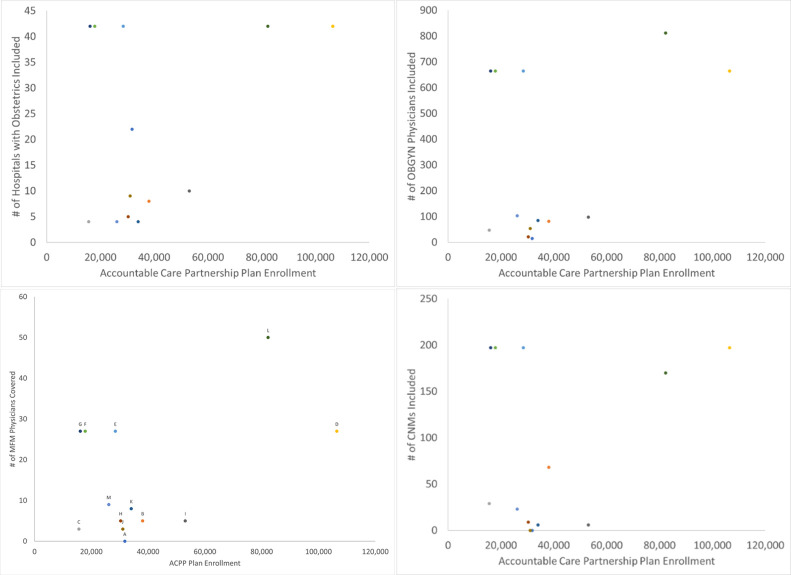
Inclusion of maternity care providers versus ACO enrollment for ACPP ACOs. a. Hospital Inclusion vs. ACPP ACO Enrollment; b. OBGYN Inclusion vs. ACPP ACO Enrollment; c. MFM Inclusion vs. ACPP ACO Enrollment; d. Midwife Inclusion vs ACPP ACO Enrollment. **Notes:** Each point in the figure represents a single ACPP ACO. ACPP enrollment based on 2018 report by the Center for Health Law and Economics, University of Massachusetts Medical School [28].

Finally, we examine variation in provider inclusion in ACPP ACOs by enrollment. Fig 2 displays the number of each type of provider (i.e., OB/GYNs, MFMs, CNMs, hospitals with obstetric department) included in the ACO compared to the number of enrollees for each ACPP ACO. Generally, the number of included providers increases with increasing enrollment. However, for smaller ACOs there is variation, with some plans including substantially more providers than others. One ACPP ACO did not include any MFM specialists in the provider directory. In some ACPP ACOs associated with the same managed care insurer, identical maternity care providers are included despite differing enrollment in the ACOs.

## Discussion

First introduced in 2011, Medicaid ACOs are a relatively new value based payment model, and there is limited prior research characterizing the breadth of provider networks in these ACOs [40]. In this analysis of Medicaid ACOs in Massachusetts, we found substantial variation in maternity care clinician inclusion in Medicaid ACOs, both across and within ACO type. Although some ACOs had broad maternity care clinician and hospital inclusion, others had few or no clinicians–specifically CNMs–included and/or included a limited number of hospitals. For patients with Medicaid, which covers a large proportion of births [41], ensuring adequate access to maternity care is important.

The few prior studies on Medicaid ACOs in maternal health care show Medicaid ACO participation resulted in an improvement of the timeliness of prenatal care initiation in the first trimester but did not increase the total receipt of adequate prenatal care [42]; state Medicaid ACO adoption is also associated with a decrease in hospital costs and cesarean birth rates [43]. A larger body of literature on ACOs focuses on spending outcomes of Medicare ACO implementation [1, 3, 4, 44–46]. In Medicare, several studies find that ACO spending outcomes depend on specialist composition of the ACO [47, 48]. In Medicare ACOs, a previous study found that two-thirds of specialist office visits in a Medicare ACO were with providers outside of the ACO network [49]; previous research has found that referrals are relatively common during prenatal care [50]. However, Medicaid ACO networks differ significantly from Medicare ACOs in that Medicare ACO enrollees can see any specialist that accepts Medicare, which is the vast majority of specialists [51], whereas Medicaid ACOs such as the Massachusetts ACPP Medicaid ACOs may limit specialist coverage to only providers included within a smaller provider network. Although these provider networks must meet state regulatory standards, this does not necessarily mean that the network is adequate to ensure access to care [10]. Despite these Massachusetts Medicaid ACO provider networks all presumably meeting state regulatory standards, we find that there continue to be large differences in maternity care provider inclusion [19]. Although Massachusetts, like 85% of states with comprehensive Medicaid managed care, uses supplemental payments to insurers to offset delivery costs, Medicaid ACOs may still have incentives to use provider networks as a mechanism to either attract or avoid enrollees who are pregnant [52].

Another important finding is that some Medicaid ACOs did not include any CNMs, and the PCACOs did not easily identify any included CNMs. CNMs attend over 17% of births in Massachusetts, including almost 20% of births covered by Medicaid [53]. Midwifery care has been shown to have equal or better outcomes for low-risk pregnancies compared to physician-led care [54–57], and the availability of this care may be particularly important for Medicaid enrollees. The midwifery model of care emphasizes psychosocial support, taking into account individual life circumstances, and supporting pregnancy and birth as normal, physiologic events [58]; as such, ensuring access to this type of maternity care clinicians in Medicaid ACOs is an important policy priority [59].

ACPPs that had high numbers of member enrollment had correspondingly high levels of provider and hospital inclusion. However, among plans that had lower member enrollment, provider and hospital inclusion varied widely. MFMs are crucial for managing maternity care for high-risk patients, yet fewer than 10 MFMs were included in a majority of ACPP ACOs, particularly in ACPP ACOs with low member enrollment. This has important implications regarding access to care for Medicaid insured individuals. As many ACOs with low levels of enrollment are located outside of major metropolitan areas, a smaller network could lead to increased distances to the nearest provider, as well as fewer options in selecting a specific clinician. Maternity care availability (including providers and insurance coverage) in rural areas is critical for ensuring adequate perinatal care, and reduced access is an ongoing issue throughout the nation [60].

The study had several limitations. First, given the known limitations of online provider directories [61, 62], our primary data source may not provide an accurate representation of the hospitals and maternity care clinicians included by each ACO. Of particular interest for the accuracy of provider listings, the PCACO plans included more OB/GYNs than the total number of OB/GYNs practicing in the state according to external estimates [35]. A previous provider directory review report by Centers for Medicare and Medicaid Services found 45% of provider locations listed in Medicare ACO directories were inaccurate [62]. Of these inaccuracies, a substantial number resulted from listing providers that did not practice at the published location. This report also highlighted that a large number of providers listed as accepting new patients were not actually accepting new patients, potentially further inflating directory offerings. Other work has found that over half of specialists and PCPs listed in the directory for Medicaid ACOs in Oregon did not actually see patients in that plan [63]. For CNMs in particular, the PCACO directory did not list specific clinicians (who were CNMs) and included a variety of non-obstetric and obstetric practices, which we did not include in the count. In spite of these limitations to provider directories, this is a key way in which enrollees interface with insurance plans [64], and the breadth of the provider network is an important factor in choosing an insurance plan [65, 66]. Second, PCACOs use the full Massachusetts Medicaid specialist network, but it is not clear from the available directory whether all of these specialists accept Medicaid; additionally, it is not clear whether they specifically accept patients from the PCACOs. In Massachusetts, a study using health insurance claims data showed that 92.6% of specialists treated at least one Medicaid patient in 2011 [67]. However, previous research using phone audit methods has shown lower rates of appointment availability for Medicaid patients, with a 3.3-fold lower rate of being able to schedule a specialty appointment compared with private insurance [68]. Third, although a newly approved extension of Medicaid ACOs in Massachusetts requires the collection of race and ethnicity data and will eventually require ACOs to report on inequities in care receipt and outcomes by race and ethnicity [69], there are currently no public data available to identify Medicaid ACOs that serve a large proportion of birthing people of color. Therefore, we were not able to determine how people of color in ACOs in particular may experience access to and choice of maternity care clinicians and hospitals.

## Conclusion

In this study, we examine variation in the maternity care provider inclusion in Medicaid ACOs in Massachusetts, which is critically important for access to care for Medicaid enrollees. We find substantial differences across ACO types and within ACO type. Understanding the role of maternity care provider inclusion in Medicaid ACO contracts and Medicaid risk adjustment and payment policy may be an important area of future research to understand incentives of Medicaid ACOs that improve maternal health care and health outcomes. Additionally, measuring the quality of included obstetric clinicians and hospitals, particularly for maternal healthcare, is an important area for ensuring equitable health care for Medicaid enrollees. As Medicaid ACOs expand nationally, ensuring that maternal healthcare is an area of focus–including sufficient and equitable access to high quality maternity care providers–will be important to ongoing efforts to improve maternal health outcomes.

## Supporting information

S1 Table Source of provider directories for MA Medicaid ACOs. Note: Directories were accessed in December 2020/January 2021. Links updated as of November 2022. Due to a change in insurer ownership, BMC HealthNet provider directories are no longer available online.
(DOCX)
