# Supplementary material for: Maternity care clinician inclusion in Medicaid Accountable Care Organizations
Source: PLoS One. 2023 Mar 8;18(3):e0282679. doi: 10.1371/journal.pone.0282679 (PMC9994708; doi:10.1371/journal.pone.0282679)
Supplement: S1 Table — Note: Directories were accessed in December 2020/January 2021. Links updated as of November 2022. Due to a change in insurer ownership, BMC HealthNet provider directories are no longer available online. (DOCX) [file pone.0282679.s001.docx]

**S1 Table. Source of provider directories for MA Medicaid ACOs**

| **ACO Type** | **Source** | **URL** |
| --- | --- | --- |
| PCACO | Community Care Cooperative (C3) | https://masshealth.ehs.state.ma.us/providerdirectory/ |
| PCACO | Mass General Brigham | https://masshealth.ehs.state.ma.us/providerdirectory/ |
| PCACO | Steward Health Choice | https://masshealth.ehs.state.ma.us/providerdirectory/ |
| ACPP | Be Healthy Partnership | http://behealthypartnership.org/wp-content/uploads/2022/11/BeHealthyPartnershipACOProviderDirectory.pdf |
| ACPP | Berkshire Fallon Health Collaborative | https://fallonhealth.org/-/media/ACO/Documents/BFHC_Provider_Directory.ashx |
| ACPP | BMC HealthNet Plan Community Alliance | https://www.wellsense.org/members/ma/masshealth#find-a-provider |
| ACPP | BMC HealthNet Plan Mercy Alliance | https://www.wellsense.org/members/ma/masshealth#find-a-provider |
| ACPP | BMC HealthNet Plan Signature Alliance | https://www.wellsense.org/members/ma/masshealth#find-a-provider |
| ACPP | BMC HealthNet Plan Southcoast Alliance | https://www.wellsense.org/members/ma/masshealth#find-a-provider |
| ACPP | Fallon 365 Care | https://fallonhealth.org/-/media/ACO/Documents/365Care_Provider_Directory.ashx |
| ACPP | Allways Health Partners – My Care Family | https://allways.sapphirecareselect.com/?ci=mycarefamily |
| ACPP | Tufts Health Together with Atrius Health | https://tuftshealthplan.com/documents/microsites/tufts-health-together-with-atrius-health/tufts-health-together-with-atrius-health-provider |
| ACPP | Tufts Health Together with BIDCO | https://tuftshealthplan.com/documents/microsites/tufts-health-together-with-bidco/tufts-health-together-with-bidco-provider-director |
| ACPP | Tufts Health Together with Boston Children’s | https://tuftshealthplan.com/documents/microsites/tufts-health-together-with-boston-children-s/tufts-health-together-with-boston-childrens-provid |
| ACPP | Tufts Health Together with CHA | https://tuftshealthplan.com/documents/microsites/tufts-health-together-with-cha/tufts-health-together-with-cha-provider-directory |
| ACPP | Wellforce Care Plan | https://fallonhealth.org/-/media/ACO/Documents/WCP_Provider_Directory.ashx |

Note: Directories were accessed in December 2020/January 2021. Links updated as of November 2022. Due to a change in insurer ownership, BMC HealthNet plans are now administered under WellSense Health Plan.
